# Narrow-Leafed Lupin (*Lupinus angustifolius* L.) Seeds Gamma-Conglutin is an Anti-Inflammatory Protein Promoting Insulin Resistance Improvement and Oxidative Stress Amelioration in PANC-1 Pancreatic Cell-Line

**DOI:** 10.3390/antiox9010012

**Published:** 2019-12-23

**Authors:** Elena Lima-Cabello, Juan D. Alché, Sonia Morales-Santana, Alfonso Clemente, Jose C. Jimenez-Lopez

**Affiliations:** 1 Department of Biochemistry, Cell & Molecular Biology of Plants, Estación Experimental del Zaidín, Spanish National Research Council (CSIC), Profesor Albareda 1, Granada E-18008, Spain; elena.lima@eez.csic.es (E.L.-C.); juandedios.alche@eez.csic.es (J.D.A.); 2 Proteomic Research Department, San Cecilio University Hospital, Biosanitary Research Institute of Granada (Ibs.GRANADA), Av. Dr. Olóriz 16, Granada E-18012, Spain; soniamoralessantana@hotmail.com; 3 Department of Physiology and Biochemistry of Animal Nutrition, Estación Experimental del Zaidín, Spanish National Research Council (CSIC), Camino del Jueves, Granada E-18100, Spain; alfonso.clemente@eez.csic.es; 4 The UWA Institute of Agriculture and School of Agriculture and Environment, The University of Western Australia, CRAWLEY Perth, WA 6019, Australia

**Keywords:** 7S basic globulins, anti-inflammatory protein, antioxidant protein, cytokines, glutathione, iNOS, nitric oxide, oxidative stress, sweet lupins group

## Abstract

(1) Background: Inflammation molecular cues and insulin resistance development are some of the main contributors for the development and advance of the pathogenesis of inflammatory-related diseases; (2) Methods: We isolated and purified γ-conglutin protein from narrow-leafed lupin (NLL or blue lupin) mature seeds using affinity-chromatography to evaluate its anti-inflammatory activities at molecular level using both, a bacterial lipopolysaccharide (LPS)-induced inflammation and an insulin resistance pancreatic cell models; (3) Results: NLL γ-conglutin achieved a plethora of functional effects as the strong reduction of cell oxidative stress induced by inflammation through decreasing proteins carbonylation, nitric oxide synthesis and inducible nitric oxide synthase (iNOS) transcriptional levels, and raising glutathione (GSH) levels and modulation of superoxide dismutase (SOD) and catalase enzymes activities. γ-conglutin induced up-regulated transcriptomic and protein levels of insulin signalling pathway IRS-1, Glut-4, and PI3K, improving glucose uptake, while decreasing pro-inflammatory mediators as iNOs, TNFα, IL-1β, INFγ, IL-6, IL-12, IL-17, and IL-27; (4) Conclusion: These results suggest a promising use of NLL γ-conglutin protein in functional foods, which could also be implemented in alternative diagnosis and therapeutic molecular tools helping to prevent and treat inflammatory-related diseases.

## 1. Introduction

The outcomes from epidemiological studies have revealed that an increasing number of health problems are affecting all societies around the globe as diabetes, insulin resistance, obesity, metabolic syndrome and cardiovascular diseases [1], where they have been associated to both scarce physical activity and the ingestion of high sugar–high lipid diets in metropolitan areas [2]. In this regard, there is an increasing demand of plant proteins highly beneficial for human health to be used for foodstuffs development and production and has prompted an increasing body of research covering diverse nutraceutical aspects in a number of crop plants. There is a strong interest focused in legumes, which are an economical important source of high-quality proteins compared to other plant foods [3]. 

Interestingly, lupin seeds, and particularly seeds from the species encompassing the “sweet lupin” group have been reported to exert beneficial effects in human health [4]. Thus, the dietary consumption of lupin seed proteins might provide preventive and protective effects (also complementing the current treatments for metabolic diseases) for different human inflammatory-related diseases such as metabolic syndrome, obesity, and high blood pressure (lowering capacity), type 2 diabetes mellitus (T2DM) development and triggered by uncontrolled glycemia throughout increasing insulin resistance, familial hypercholesterolemia and cardiovascular disease [5]. Different factors or stressors promote and stimulate immune-response-mediated inflammation leading to the molecular mechanisms underlying many of these diseases including defective insulin secretion and responses, and finally to the insulin resistance which has the pancreatic tissue as the key target for this disease evolution, progressing with an uncontrolled synthesis of pro-inflammatory mediators. Among them, interleukin 6 (IL-6), interleukin 1 (IL-1), interferon gamma (INFγ), tumor necrosis factor (TNF-α), chemokines (i.e., CCL2, CCL5), reactive oxygen species (ROS) as H_2_O_2_, peroxide and superoxide anion, nitric oxide (NO) overproduction, and nitrogen intermediate molecules, as well as adhesion molecules release (i.e., ICAM-1, VCAM-1) facilitating immune system cells attraction and movement through the tissues enhancing the inflammatory response [6]. The most frequently associated stressors are oxidative stress, alterations in gut microbiota that increase lipopolysaccharides (LPS) in blood, lipotoxicity, glucotoxicity and endoplasmic reticulum (ER) stress promoting misfolded proteins that may be deposited in the islets β-cells in form of amyloids [6]; these amyloid deposits enhance inflammatory response mediated by immune cells attracted to the pancreatic tissues [7].

Thus, lowering the synthesis and/or functional role of pro-inflammatory molecules has the advance of potentiate an anti-inflammatory reaction that may also help to the inflammatory-related diseases amelioration.

Searching for naturally-occurring compounds with the potential of anti-inflammatory responses has also increased parallel to the risen number of inflammation-related diseases. Only a few studies have described the anti-inflammatory properties of some seeds-derived bioactive hydrolysates of proteins; however, even more scarce are the studies concerning legume seed compounds with these potential functional activities. Interestingly, enzymatic hydrolysates of field pea seeds showed anti-inflammatory properties at molecular level by inhibiting several inflammation mediators’ production, i.e., NO and TNFα [8]. Lunasin, a peptide derived of isolated 2S albumin that was found in soybean, as well in some cereal grains displayed great benefits related to cancer amelioration, cardiovascular disease improvement and lowering cholesterol [9]. In soybean, the anti-inflammatory properties of lunasin have been associated to its ability to suppress the NFκΒ functional pathway [10]. Seed protein hydrolysates from blue lupin were found to have the potential to inhibit phospholipase A2 and cyclooxygenase-2 enzymes that are involved in the inflammatory pathway [11]. Another further study showed the example of bioactive peptides with high homology with *Arabidopsis thaliana* 2S albumin and *Glycine max* lectin-like protein, which were associated with genes expression modulation of inflammatory molecules [12].

In this work, we have studied the anti-inflammatory properties of narrow-leafed lupin (NLL) γ-conglutin protein from mature seeds using in vitro human PANC-1 pancreatic cell-line in both, an induced inflammation model using bacteria lipopolysaccharide (LPS), and an induced insulin resistance (IR) cell model, with the aim of assessing the capability of NLL γ-conglutin to improve the oxidative stress homeostasis of cells, the inflammatory induced state and the IR improvement at molecular level by decreasing several pro-inflammatory mediators genes expression and proteins levels, as well as up-regulating of insulin signaling pathway gene expression.

## 2. Material and Methods

### 2.1. Isolation and Purification of γ-Conglutin from NLL Mature Seeds

The isolation and purification of γ-conglutin proteins from NLL was accomplished following the Czubiński et al. [13] method. Briefly, NLL seed proteins were extracted using Tris buffer pH 7.5 [20 mmol L^−1^], having 0.5 mol L^−1^ NaCl/gr defatted seeds. After sample centrifugation at 20,000× *g*, 30 min at 4 °C, the supernatant was filtered using a 0.45 μm syringe filter of PVDF. Thus, the sample was ready to be introduced in a desalting column of Sephadex G-25 medium. The desalted crude protein sample was applied to a HiTrap Q HP column (GE Healthcare) previously equilibrated with Tris buffer pH 7.5 [20 mmol L^−1^], where the proteins’ separation was possible using a linear gradient [0 to 1 mol L^−1^] of NaCl. Under these conditions, the γ-conglutin proteins were not retained on the media contained in the column. Thus, different fractions that contained γ-conglutin proteins were pooled and introduced on HiTrap SP HP column (GE Healthcare) previously equilibrated with Tris buffer pH 7.5 [20 mmol L^−1^]. γ-conglutin proteins retained in this column were eluted with a linear gradient of NaCl [0 to 0.5 mol L^−1^]. The γ-conglutin proteins were collected and directly used in the further SDS-PAGE analysis and fingerprinting characterization. The remaining protein was kept frozen at −80 °C.

### 2.2. Analysis of Purified γ-Conglutin Protein by Peptide Mass Fingerprinting

The identity proof of the purified γ-conglutin protein was achieved following peptide mass fingerprinting. Briefly, proteins (10 μg) were separated by SDS-PAGE using precast gels of 12% Bis-Tris (Invitrogen) under reduced conditions. Electrophoretic bands corresponding to γ-conglutin protein (bands 1 to 4, Supplementary Figure S1), were cut out from the gel and in-gel trypsin digested. These peptide fragments generated were subjected to desalt and concentration, to be afterward loaded onto the MALDI plate and analyzed. MALDI-MS spectra were generated in a 4700 Proteomics Analyzer (Applied Biosystems, Waltham, MA, USA), and these data were used for proteins ID validation (www.matrixscience.com).

### 2.3. SDS-PAGE and Immunoblotting

Analysis of protein extracts were made by mixing the samples sample buffer (6× concentrated) and heated during 5 min up to 95 °C. Proteins were separated by SDS-PAGE using gradient TGX gels of 4–20% acrylamide (Bio-Rad). To identify the molecular weight (MW) of separated proteins we used a MW marker for stained gels as Mark12 Unstained Standard (ThermoFisher Scientific), with a MW range between 2.5 to 200 kDa. The resolved protein bands were visualized in a Gel Doc™ EZ Imager (Bio-Rad, Berkeley, CA, USA). For immunoblotting, proteins were transferred to PVDF membranes, which afterward were blocked for 2 h at room temperature (RT) using 5% of non-fat dry milk dissolved in PBST (phosphate-buffered saline, 0.05% Tween-20). Different membranes were incubated with goat anti-TNFα (Abcam, ref. ab8348, Cambridge, UK) at 1:1000 dilution; anti-IL-1β (Abcam, ref. ab9722) at 1:500; anti-iNOS (Invitrogen, ref. PA1-036, Carlsbad, USA) at 1:1000; anti-IRS1 (Sigma-Aldrich, Ref. 06-248, Darmstadt, Germany) at 1:500; anti GLUT4 (Sigma-Aldrich, ref. 07-1401) at 1:1000; and anti-PI3K (Abcam, ref. 86714) at 1:500. All the incubations were made leaving the membranes overnight at 4 °C in constant movement. Next day, membranes were washed for 5 times with PBST, followed by incubation with horseradish peroxidase-conjugated anti-rabbit IgG (Sigma-Aldrich, ref. A9169) at 1:2500 dilution in 2% non-fat dry milk dissolved in PBST 24 for 2 h at RT. The membranes were then washed 5 times with PBST; signal development was achieved for each antibody by incubation with ECL Plus chemiluminescence following the manufacturer’s instructions (Bio-Rad). The reactive bands in the membranes were detected by exposure to C-DiGit Blot Scanner (LI-COR).

### 2.4. Cell Culture and Treatment

The PANC-1 pancreatic cells were grown in poly-L-lysine-coated 75 cm^2^ flasks (∼2.5 × 10^6^ cells/mL) in Dulbecco’s modified Eagle’s medium (DMEM) supplemented with heat-inactivated fetal bovine serum (10%) and 2 mM glutamine, all at final concentration, in a 5% CO_2_/95% humidified atmosphere at 37 °C.

The pancreatic cells were maintained by serial passage in culture flasks and used in the experimental studies when the exponential phase was reached. Cells were grown to confluence and the monolayer culture was washed two times with phosphate-buffered solution (PBS, Sigma). The cells were then treated with trypsin-EDTA (Lonza) at 0.25% for 10 min. After 5 min centrifugation at 1000× *g* and two times PBS washing, PANC-1 cells were collected. Afterward, cells counting and viability assessment were achieved by using a Countess II FL Automated Cell Counter (Thermo Fisher) at both, the initial and final step of each experiment. Viability of cells was higher than 95%. Cell cultures were stablished at 80% of confluence and treated with LPS (1 μg/mL) for 24 h. PANC-1 cells were challenged with purified γ-conglutin protein for 24 h alone or in combination adding LPS. Aliquots of γ-conglutin protein stored at −20 °C in PBS were thawed just before use and dissolved in culture media to target concentrations and to be added to the cultures. After treatment, cells were harvested for further analyses.

### 2.5. MTT Assay for Cell Viability

Cell viability was evaluated using 3-(4,5-dimethylthiazol-2-yl)-2,5-diphenyltetrazolium bromide (MTT) following the manufacturer’s instructions (Roche). Briefly, 96-well microtitre plates were inoculated at a density of 1 × 10^3^ PANC-1 cells per well in 300 μL of growth media. Plates were incubated overnight under 5% CO_2_ in humidified air to allow the cells to adhere to the wells. After incubation, cells were treated for 24 h with either LPS or γ-conglutin protein, and washed three times with PBS in order to prevent any interfering issue because of the phenolic compounds when making the MTT assay. A volume of 200 μL of free red-phenol DMEM containing 1 mg mL^−1^ of MTT was added to the cells, and these were incubated for 3 h. Metabolically active viable cells are able to convert MTT into formazan crystals (purple color), and the former compound was solubilized with 200 μL of DMSO to absorb at 570 nm (test) and 690 nm using a iMark microplate reader (Bio-Rad, USA).

### 2.6. Insulin Resistance PANC-1 Cell Model and Glucose Uptake

Culture PANC-1 control cells were seeded in DMEM supplemented with 10% (v/v) FBS, using 96-well microtiter plates under standard conditions (5% CO_2_ and 37 °C in humidified air), and a density of 2 × 10^4^ cells per mL in 200 mL. Optimal dose of insulin and treatment time as requisite to establish insulin-resistant IR_PANC-1 (IR-C) cells. Cells display reduced glucose uptake, and this is one of the main feature of the insulin resistance impaired glucose uptake since decreasing cells responses to glucose uptake to increasing levels of insulin. Thus, the cell culture was separated into two groups having six independent replicates per each group: (1) Cultured cells in 200 μL complete medium (control cells, group C); (2) Treated cells with insulin (10^−5^ to 10^−9^ nmol L^−1^) when the cells became adherent (group IR-C). These PANC-1 cells were then cultured for 24, 48, and 72 h and the concentration of glucose in the media was measured using the glucose oxidase method (Abcam, UK). The concentration required to stablish IR-C PANC-1 cells was 10^−7^ nmol L^−1^ and cultured for 24 h. At this IR stage, it was evaluated whether cells were sensitive to insulin and to evaluate whether γ-conglutin protein can improve the insulin-dependent glucose uptake capacity of IR-C PANC-1 cells. Thus, these cells were separated in three groups, each one with six replicates: The control group (C), IR-C and the IR-C + γ-conglutin groups. After 24 h, 2 μL of culture supernatant was collected from each sample and glucose concentration was determined as described above. Cultures of IR-C cells were stablished to 80% confluence and challenged with γ-conglutin protein for 24 h. After the treatments, the cells were harvested for further analyses.

### 2.7. Quantitative Real-Time PCR

GLUT-4, IL-1β, iNOS, IRS-1, PI3K and TNFα mRNA expression were assayed by mean of Real-time quantitative PCR for each experimental group. Total RNA was isolated from group C using the RNeasy Tissue RNA isolation kit (Qiagen, Hilden, Germany). First strand cDNA was synthesized using a High-Capacity cDNA Archive Kit (Applied Biosystems, Waltham, MA, USA). cDNA was prepared, diluted and subjected to real-time polymerase chain reaction (PCR), and amplified using TaqMan technology (LightCycler 480 quantitative PCR System, Roche, Basel, Switzerland) for gene expression assays. Primers and probes were used from the commercially available TaqMan Gene Expression Assays [IRS-1: Assay ID Hs00178563_m1, GLUT-4: Hs00168966_m1, PI3K: Hs00898511_m1, TNFα: Hs01555410_m1, IL-1β: Hs01075529_m1, iNOS: Hs00174128_m1, respectively]. Gene expression levels relative changes were assessed using the 2^−ΔΔCt^ method. The cycle number where the transcripts were detectable (CT) was normalized to the cycle number of β-actin detection as housekeeping gene (Assay ID: Hs99999903_m1, Applied Biosystems), and referred to as ΔCT, where the relative mRNA levels are presented as unit values of 2^∧ [CT (β-actin)–CT (gene of interest)]^, and displaying CT as the threshold cycle value. This parameter was defined as the fractional cycle number at which the target fluorescent signal passes a fixed threshold above baseline. PCR efficiency was assessed by TaqMan analysis on a standard curve for targets and endogenous control amplifications, which were highly similar.

### 2.8. ELISA Assays for INFγ and Cytokines Quantification

The cell cultured were prepared by cell counting and plated in six-well plates including 10^6^ cells per well, and a duplicated well per group. After 24 h incubation, the media from treated culture was eliminated and cells were washed with PBS at 4 °C. To achieve proteins extraction, temperature of the plates was kept closely to 4 °C by placing these on ice, thus avoiding the denaturation of cytokines. One hundred microliters of buffer (150 mM sodium chloride, 1% NP-40, 50mM Tris pH 8) was added to each well and supplemented with 1 μL of protease inhibitor (Sigma) for 15 s. Scraped cells from the bottom of the wells were transferred to microcentrifuge tubes. These tubes were centrifuged at 12,500× *g* for 15 min at 4 °C. After this step, every supernatant was collected and diluted to a 1:4 ratio used for the ELISA quantification test of INFγ, IL-6, IL12p70, IL-17, and IL-27 (Diaclone). Data were statistically analyzed using the *t*-test.

### 2.9. Antioxidant Enzymatic Activity Assays

The cell cultures were prepared and after 24 h of incubation of the treated culture, growing media was removed, and cells washed with PBS at 4 °C. Cells from C, IR-C and IR-C cultures challenged with γ-conglutin protein were collected and used for the enzymatic activity assessment of SOD and catalase, as well as the GSH measurement (Canvax, Córdoba, Spain), following manufacturer’s instructions. Data were analyzed by the statistical t-test.

### 2.10. Determination of Intracellular ROS and Nitric Oxide (NO)

C and IR-C cell cultures, challenged or not with γ-conglutin protein, were used for proteins extraction and following company instructions either for control or treatment samples (EMD Millipore, USA). A total proteins quantity of 25 μg was loaded onto polyacrylamide gels at 12% for proteins separation by SDS-PAGE. Achieved this step, proteins were transferred to PVDF membranes to be used for protein oxidation detection by using The OxyBlot™ Kit (EMD Millipore, Burlington, MA, USA) according to the manufacturer’s instructions, This kit was used for the detection of carbonyl groups present into proteins because the proteins reaction with ROS. Measurements were developed at 485 nm and 530 nm excitation and emission wavelengths, respectively.

The total amount of NO, including nitrite/nitrate content, was measured using a commercial assay kit [ab65328, Abcam, Cambridge, UK] from C and IR-C culture cells before and after γ-conglutin protein challenges. Briefly, samples including every experimental group were deproteinized according to the manufacturer’s instructions. An equal amount of sample (30 μL) and standards were loaded into 96-well microtiter plates. Nitrate reductase, enzyme cofactor and assay buffer were added following a 1 h of incubation at RT with Enhancer, Griess Reagent R1 and Griess Reagent R2. Just after incubation, samples were used to measure absorbance at 540 nm with an i-Mark microplate reader (Bio-Rad, USA). The value of the blank control (medium without cells) was subtracted to the samples’ values. Total nitrite/nitrate concentrations were calculated by using a standard curve.

### 2.11. Statistical Analysis

Data obtained from each experimental were expressed as means ± standard deviation (SD). Experimental assessment was developed at least three times. The one-way variance analysis was implemented using SPSS statistical software (SPSS Inc., Chicago, IL, USA). Statistical significance of differences (*p* < 0.05) in the analyzed data was evaluated with the use of SPSS software by analysis of variance and Dunnett analysis afterward.

## 3. Results and Discussion

### 3.1. Isolation and Purification of the NLL Anti-Inflammatory γ-Conglutin Protein

The γ-conglutin protein extraction, isolation and purification were accomplished following the methodology from Czubinski et al. [13] using mature NLL seeds as starting material. A representative SDS-PAGE is shown in supplementary Figure S1. The sample from γ-conglutin purification went to the electrophoretically separation under reduced conditions; several different electrophoretic bands were found for this protein. The most abundant forms were the separated α and β subunits, followed by the unreduced γ-conglutin (α + β subunits) and the uncleaved γ-conglutin precursor [14]. The γ-conglutin monomer is integrated by two subunits (α + β) linked by a single disulphide bridge, which is highly resistant to be broken under reducing conditions due to the structure of the monomeric protein [15].

The expected MW of the γ-conglutin monomer from these sequences is ∼45 kDa. After reduction of the disulphide bridge, two electrophoretic bands of 30 kDa (α-subunit) and 17 kDa (β-subunit) were detected, in addition to a ∼56.0 kDa band corresponding to the uncleaved γ-conglutin precursor (Supplementary Figure S1, Supplementary Table S1). The purity of this isolated protein assayed by SDS-PAGE under reducing conditions (Supplementary Figure S1) reached a 95%.

In order to identify the different bands showed in the SDS-PAGE gel corresponding to the isolated and purified γ-conglutin (Supplementary Figure S1), we performed an in-gel tryptic digestion of the cut bands, and these were subjected to separation of the peptides and MS-based analysis. The peptide mass data generated was searched against the MS protein sequence database enabled the unambiguous identification by mass peptide fingerprinting as γ-conglutin (NLL 7S-basic globulin) (Supplementary Table S1).

### 3.2. Cell Viability Assessment of the PANC-1 Cells Treated with γ-Conglutin Protein

In this study, we assessed the viability of PANC-1 cells under treatment of the γ-conglutin protein and the potential cytotoxicity of this protein. In order to evaluate whether inflammation inductor LPS and γ-conglutin produce cell cytotoxicity effects, the viability MTT assay was achieved on PANC-1 cells under separate treatments with LPS adding γ-conglutin, at increasing concentrations to complete the conditions of DMEM culture medium + FBS + antibiotic for 24 h. The LPS plus γ-conglutin had no significant (*p* > 0.05) effects on cell viability (Supplementary Table S2), when compared with the control (untreated) group. The cell cultures used as positive control lacked LPS and γ-conglutin protein. In order to complete the usefulness of the γ-conglutin protein study, trypan blue staining was also used for assessing PANC-1-pancreatic cells viability after treatment with LPS (1 μg/μL) and increasing concentrations (from 10 to 50 μg) of γ-conglutin for 24 h, finding significant differences (*p* < 0.05) in cell viability after 24 h of incubation only at 50 μg compared to the control (Supplementary Table S2). 

Furthermore, a parallel study was made to assess the cell viability and cytotoxicity of increasing concentrations of insulin in order to know whether an insulin resistance model could be performed in PANC-1 pancreatic cells and to know the actual insulin concentration that should be used to stablish the model. An MTT assay was developed on PANC-1 cell finding that an important change in the percentage of viability was induced for insulin concentrations higher than 10^−7^ nmol L^−1^ (Supplementary Table S3). Afterward, IR-C cells were assayed for viability using MTT kit when performed the addition of γ-conglutin protein for 24 h. No significant (*p* > 0.05) effect on cell viability (when treated with 25 μg of γ-conglutin protein) (Supplementary Table S4) was found after comparison with unchallenged IR-C group. When insulin was added alone (in the absence of γ-conglutin), these samples were used as a positive control. We also performed the cell viability assessment using trypan blue exclusion in IR-C pancreatic cells treated with increasing concentrations of this protein for a period of 24 h. No cell viability differences were found after 24 h of incubation in the presence of γ-conglutin. 

These results suggest that γ-conglutin do not affect to the PANC-1 pancreatic cell integrity in both, the induced (LPS treatment) inflammation and the IR-C cell models.

### 3.3. Effect of γ-Conglutin Protein on the Inflammatory Process

Inflammatory-related illnesses as metabolic syndrome, T2DM, obesity and cardiovascular diseases are well known to be developed and chronically associated to a continuously sustained inflammatory state. Among different mechanisms hidden in the inflammatory-based diseases, different molecules namely stressors affect functional pancreatic tissues physiology, particularly β-islets, promoting the course of pathology, which also of course mainly depend of particular genetic backgrounds and environmental factors [16].

**Figure 1:**
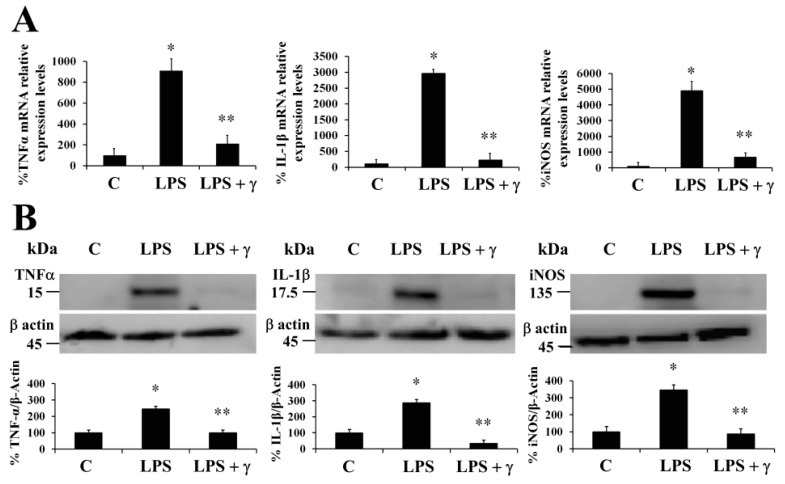
Narrow-leafed lupin (NLL) γ-conglutin decreases the mRNA expression and protein levels of TNFα, IL-1β, and iNOS on lipopolysaccharide (LPS)-induced inflammation pancreatic cells. PANC-1 cells were incubated for 24 h with LPS alone or γ-conglutin + LPS. (**A**) The bar graph shows mRNA levels determined by real-time RT-qPCR of TNFα, iNOS, and IL1β. (**B**) The bar graph shows protein levels determined by immunoblotting of TNFα, iNOS, and IL1β. Average value from triplicate experiments of each biomarker were relativized to the average value of their housekeeping actin protein in control samples. Then, average values from challenge experiments (calculated in the same way than controls) are relativized to these from their respective control values previously calculated. Data represent mean ± SD from three independent experiments. C: Untreated control culture cells; LPS: LPS-treated culture cells; LPS + γ: LPS + γ-conglutin challenge. *p* < 0.05 represents statistically significant differences associated with each figure. p* < 0.05 LPS *versus* C; p** < 0.05 LPS + γ-conglutin *versus* LPS. Challenges were made with LPS and/or γ-conglutin protein at 1 μg/mL and 25 μg, respectively.

Nowadays, there is an increasing number of diabetes associated to obesity named “Diabesity epidemic”, which is frequently coincidental with a pancreatic islet cells failure unable to generate enough amount of insulin and/or a developed decreasing sensitivity to insulin by tissues able to metabolize glucose. During the establishment of T2DM, sustained high levels of glucose may lead to organ damage, which is mediated by pancreatic β-cells tissue damage, and the enhancement of immune system inflammatory response because the synthesis and release of pro-inflammatory mediators as cytokines and chemokines (cells chemotactic factors). These processes create feed-forward progressive steps that further increases immune system cell content, promoting a chronic inflammatory state [17]. Thus, increasing levels of multiple factors as IL-1β, TNFα, and iNOS are important contributors for the development of inflammation since IL-1β-mediates β-cell dysfunction during the development of T2DM, while are able to activate the expression of iNOS with the result of an exacerbate synthesis of NO, promoting the up-regulation of pro-inflammatory genes [18]. In this regard, we evaluated the ability of γ-conglutin protein to modulate the mRNA levels of genes of pro-inflammatory mediators as potential anti-inflammatory targets (TNFα, IL-1β, and iNOS mRNA) in PANC-1 cells (Figure 1). Induced inflammatory state by LPS was significantly inhibited (*p* < 0.05) by γ-conglutin proteins at mRNA expression level in PANC-1 [−694, −2733, and −4208–fold, respectively, *versus* LPS treated culture cells] (Figure 1A). No statistically significant differences were observed in IL-1β cytokine, TNFα, and iNOS mRNA levels (*p* > 0.05) when challenges were performed with γ-conglutin + LPS as compared to the control group (Figure 1A). These results highlight the potential implications of γ-conglutin to decrease the pro-inflammatory capacity in PANC-1 cells by decreasing cytokines and iNOS genes expression levels, thus supporting the inflammatory process amelioration at molecular level. In this study, this lowering in the cellular pro-inflammatory capacity could be the result of the antioxidant capacity of γ-conglutin since changes in GSH levels, SOD and catalase activities was shown, helping to keep redox homeostasis in T2DM and other inflammatory-dependent diseases also affected by the oxidative stress [19]. On this line, the above results on PANC-1 pancreatic cells are in agreement with previous studies that shown a similar reduction in the expression levels of iNOS and IL-1β mRNA in T2DM blood culture [20].

It is well established that systemic production of IL-1β at local tissues plays a fundamental role in the progression of pancreatic dysfunction as β-cell apoptosis in T2DM. The advance of this disease is facilitated by a continued production of inflammatory molecular mediators that would have an initial development stage and further progression promoted by TNFα-/IL-1β-mediated iNOS synthesis and NO production [21]. We have also demonstrated that NLL γ-conglutin can reverse this state by decreasing the levels of TNFα, IL-1β and iNOS functional protein levels in PANC-1 [−158, −144, and −164–fold, respectively, *versus* LPS treated culture cells] (Figure 1B, Supplementary Table S5), while no statistically significant differences (*p* > 0.05) were observed in TNFα, IL-1β and iNOS protein levels when challenges were accomplished with γ-conglutin (LPS + γ) when compared to the control group (Figure 1B).

### 3.4. γ-Conglutin Protein Inhibits the Production of Different Cytokines and Pro-Inflammatory Mediators

Physiological circulating levels of cytokines have important implications in the functional regulation of pancreatic β-cells, although these produce different cytokines itself in response to physio-pathological states, playing also important roles in its own β-cells function [18]. When insulin resistance is stablished, increasing production of dangerous pro-inflammatory circulating mediators is also stablished. During the T2DM state progression, this non-physiological condition is characterized by an imbalance pro-inflammatory cytokines and mediators profile, led by the β-cell dysfunction and T2DM sustainable situation, which on the other hand, is based on the crosstalk among cytokines in pancreatic β-cells and immune tissues [22]. Thus, restoring the balance back to the increased levels of protective plasma circulating and β-cells cytokines could prevent and promote the treatment of this β-cell dysfunctional statement, and for extension the T2DM progression. 

**Figure 2:**
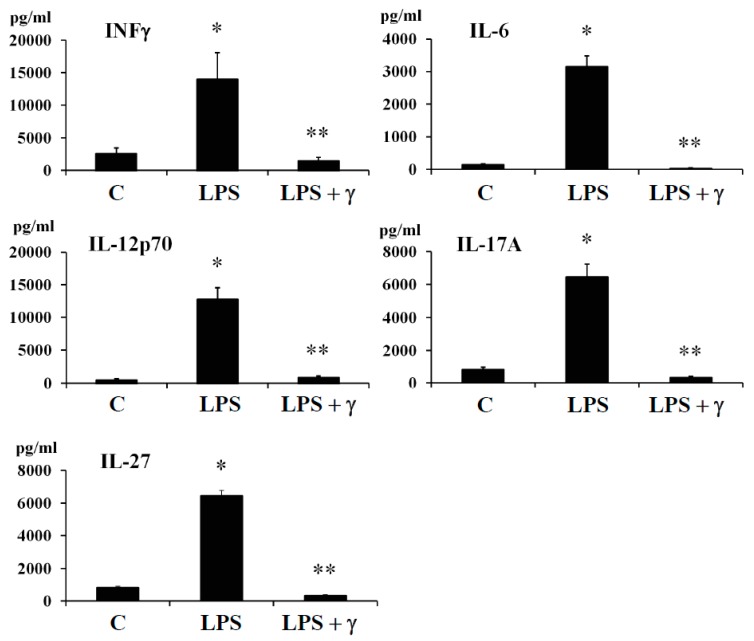
Effect of NLL γ-conglutin on the protein levels of pro-inflammatory cytokines. PANC-1 cells were incubated for 24 h with LPS alone, or γ-conglutin + LPS. The bar graph shows protein levels determined by ELISA of INFγ, IL-6, IL-12, IL-17, and IL-27. Data represent mean ± SD from three independent experiments. C: Untreated control culture cells; LPS: LPS-treated culture cells; LPS + γ: LPS + γ-conglutin challenge. *p* < 0.05 represents statistically significant differences associated with each figure. p* < 0.05 LPS *versus* C; p** < 0.05 LPS + γ-conglutin *versus* LPS. Challenges were made with LPS and/or γ-conglutin at 1 μg/mL and 25 μg, respectively.

In this regard, we evaluated by ELISA method the potential anti-inflammatory effects of γ-conglutin protein through its capacity to modulate the amount of important pro-inflammatory mediator as INF-γ and cytokines (IL-6, IL-12p70, IL-17A, and IL-27) in both, an induced inflammation model (Figure 2, Supplementary Tables S5 and S6), and in an IR-C cell model (Supplementary Figure S2, Supplementary Tables S5 and S6) using PANC-1-pancreatic cells. Levels of INFγ and the above cytokines were assessed under basal conditions, after cell treatment with LPS, by challenging the cell culture with γ-conglutin protein after LPS and by adding LPS + γ-conglutin together, or alternatively with γ-conglutin after IR-C model is stablished (as explained in material and methods, Section 2.6). The protein levels of INF-γ and cytokines (IL-6, IL-12p70, IL-17A, and IL-27) significantly (*p* < 0.05) augmented (several-fold) after LPS challenges [LPS: +11335, +2979, +12127, +5632 and +5676-fold *versus* C, respectively] (Figure 2, Supplementary Table S6); and IR-C model [+8994, +1881, +11592, +5553, +5231-fold *versus* C, respectively] (Supplementary Figure S2, Supplementary Table S6) whereas the LPS + γ -conglutin protein challenges showed a significant reduction (several-folds) in protein levels [LPS: −256, −1849, −11786, −5339 and −6100-fold *versus* LPS treated cells, respectively], and IR-C model [−8644, −1839, −11409, −5659 and −5339-fold *versus* IR-C cells, respectively] (Supplementary Figure S2, Supplementary Table S5). These results are in agreement with these obtained from RT-qPCR, where reduced mRNA and proteins levels of the pro-inflammatory mediators TNFα, IL-1β, and iNOS were found in PANC-1-cells culture (Figure 1A, Figure 1B, Supplementary Tables S5 and S6). No statistically significant differences (*p* > 0.05) were observed in INF-γ and cytokines (IL-6, IL-12p70, IL-17A, and IL-27) protein levels when challenges were accomplished with γ-conglutin (LPS + γ) after comparison to the control group (Figure 2, Supplementary Figure S2).

**Figure 3:**
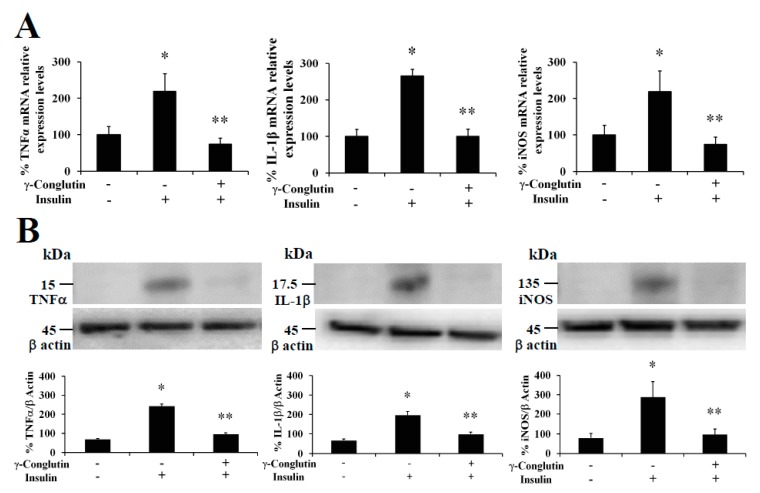
NLL γ-conglutin decreases the mRNA expression and protein levels of TNFα, IL-1β and iNOS on an insulin-resistance IR-C cell model. Control PANC-1 cells, and IR-C pancreatic cells were cultured for 24 h alone, or the former culture with γ-conglutin. (**A**) The bar graph shows mRNA levels determined by real-time RT-qPCR of TNFα, iNOS and IL1β. (B) The bar graph shows protein levels determined by immunoblotting of TNFα, iNOS and IL1β. Average value from triplicate experiments of each biomarker were relativized to the average value of their housekeeping actin protein in control samples. Then, average values from challenge experiments (calculated in the same way than controls) are relativized to these from their respective control values previously calculated. Data represent mean ± SD from three independent experiments. Control: Untreated control PANC-1 culture cells; IR-C: insulin resistant culture cells; IR-C + γ: IR-C + γ-conglutin challenge. *p* < 0.05 represents statistically significant differences associated with each figure. p* < 0.05 IR-C *versus* control PANC-1 cells; p** < 0.05 IR-C + γ-conglutin *versus* IR-C. Challenges were made with 25 μg of γ-conglutin.

Currently, scarce studies have showed results concerning the anti-inflammatory effects of plant peptides, usually promoted by the modulation of the balance regulation of pro-inflammatory interleukins, INFγ, TNFα and NO. In the case of studied soybean peptides, these inhibited mRNA iNOS expression levels and TNFα and NO production, while also reduced the pro-inflammatory enzymatic activity of COX-2 in LPS-induced macrophages [8]. Moreover, lunasin was shown to reduce the ROS production in macrophages induced by LPS while inhibiting the release of IL-6 and TNFα [11,12]. In this regard, we demonstrated that NLL γ-conglutin protein lowered the pro-inflammatory mediators’ levels assayed. This anti-inflammatory capacity would be capable to manage the diseases developmental states promoting feed-forward process for the establishment of these chronic inflammatory-derived diseases as T2DM. Thus, lupin γ-conglutins may be capable to promote the improvement from the detrimental effects of several inflammatory molecular developments as follows:

(i) Lipotoxicity as a sustained high lipid diet induces the production of IL-1β, IL-6, which β-cells continued exposure induces exacerbate synthesis and release of ROS, while secretion of insulin is also inhibited. This combination promotes the apoptosis of the pancreatic β-cells [23]. Based in our research, challenging pancreatic β-cells with γ-conglutin decreased the mRNA expression of IL-1β, and protein levels of IL-1β and IL-6 in LPS-induced inflammation [LPS + γ: −2749; −146 and −1100-fold *versus* LPS treated cells, respectively] (Figure 1; Figure 2, Supplementary Table S5); and IR-C model [IR-C + γ: −177; −97 and −1849-fold *versus* IR-C cells, respectively] (Figure 3, Supplementary Figure S2, Supplementary Table S5). 

(ii) Apoptosis of islets β-cells prompted by IL-1β and INFγ is stimulated by endoplasmic reticulum stress [24]. In this regard, β-cell apoptosis is also activated by the join action of INFγ and TNFα, together with the activation of Ca^2+^ channels. This situation induces the NO synthesis and consequently the endoplasmic reticulum stress pathway activation [25], leading to caspases activation and mitochondrial dysfunction [26]. In this concern, γ -conglutin may be able to prevent these mechanisms by suppressing the TNFα, IL-1β and INFγ mRNA and protein levels (Figure 1, Figure 2 and Figure 3, Supplementary Figure S2, Supplementary Tables S5 and S6).

(iii) The synergistic action of IL-1β + INFγ, or even IL-1β + INFγ + TNFα cytokines in pancreatic tissues increases NO production as consequence of direct increasing of iNOS, resulting in islet β-cell destruction [27]. We have shown that mRNA expression levels of TNFα and IFNγ (apoptosis mediated molecules) were lowered after treatment with γ-conglutin (Figure 1, Figure 2 and Figure 3, Supplementary Figure S2, Supplementary Tables S5 and S6), which may have a positive effect on the survival of islet β-cells [28]. 

(iv) IL-12 mRNA expression levels are increased by the effect of INFγ, while IL-12 promote signaling positive feed-back effect for raising levels of INFγ [29]. The γ-conglutin protein reduction effect of IL-12 mRNA levels (Figure 2, Supplementary Figure S2, Supplementary Tables S5 and S6) may decrease INFγ levels and its negative inflammatory effects.

(v) Important inflammatory cytokine, IL-17A, involved in the T2DM progressing, is able to induce ROS production, which also greatly affects to insulin resistance. A join action from IL-17 and INFγ acts as diabetes chronic state development [30]. Overall, IL-17A has pleiotropic functional effects comprising synthesis of IL-6 and TNFα, and chemokines (chemotaxis effect) on a diversity of cells [31]. Thus, the lowering of the IL-17 protein level (Figure 2, Supplementary Figure S2, Supplementary Tables S5 and S6) might reduce pro-inflammatory effects of IL-6 and TNFα (Figure 2, Supplementary Figure S2, Supplementary Tables S5 and S6), avoiding islet β-cell apoptosis and the recruitment of immune cells to local tissues, enhancing feed-forward mechanism of inflammation progression in islets [32] as preventive action for inflammation based T2DM progression. 

### 3.5. γ-Conglutin Reverses the Insulin Resistance through Inflammation Amelioration while Improving Insulin Signalling Pathway in Pancreatic IR-C Cells

Insulin resistance is another consequence of a sustained inflammation, which has been observed in several pathophysiological processes, including metabolic disorders as hyperinsulinemia, hyperglycemia, and hypertriglyceridemia, being IR also an important cause of pre-diabetes establishment and T2DM development and obesity [33], affecting to different insulin target organs. Thus, amelioration of IR by NLL γ-conglutin may constitute a major approach to prevent and treat these metabolic disorders.

**Figure 4:**
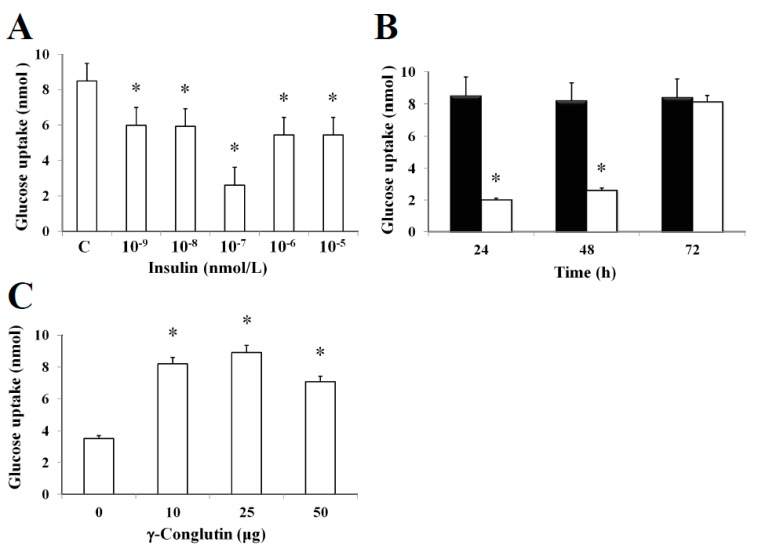
Insulin-resistant IR_PANC-1 cell model and glucose consumption promoted by γ-conglutin. (**A**) Increasing concentrations of insulin from 10^−9^ to 10^−5^ nmol/L showed that cell culture did uptake the lower level of glucose at 10^−7^ nmol/L in comparison to C cell culture, taking this concentration as the level of insulin where cells acquired the resistance state. (**B**) C cells were cultured for 24, 48 and 72 h, testing the glucose uptake of cultures including 10^−7^ nmol/L (white bars), in comparison to control C cells (black bars). In these assays were showed that insulin resistance state is preserved for 48 h. p* ˂ 0.05 IR-C *versus* C. (**C**) Glucose consumption by IR-C cells promoted by γ-conglutin at 0, 10, 25 and 50 μg was assayed after 24 h of culture. Values are shown as the mean ± SD from three independent experiments. *p* < 0.05 represents statistically significant differences associated with each figure. p* < 0.05 treated cells (μg) *versus* control.

In this study, it was established an in vitro insulin-resistant (IR-C) cell model using PANC-1 cells to evaluate the insulin effects on glucose uptake and metabolism in IR-C cell. To evaluate glucose uptake, control cells were incubated with a range of insulin concentrations (between 10^−5^ to 10^−9^ nmol L^−1^) for 24 h (Figure 4). Following an insulin concentration of 10^−7^ nmol L^−1^, we found the most statistically significant reduction in the extracellular glucose depletion (*p* < 0.05) in comparison to control cells (without insulin treatment) (Figure 4A). The addition of 10^−7^ nmol L^−1^ of insulin promoted a time-dependent lowering (*p* < 0.05) of glucose consumption between 24–48 h when compared to control cells (Figure 4B). These results clearly showed the maintenance of the insulin resistance by IR-C cells for a period of 48 h after insulin treatment. Following 48 h, cells acquired a normal condition as control cells (C). These results are consistent with the increasing glucose uptake shown in Figure 3B after 72 h, while no statistically significant differences (*p* > 0.05) in glucose consumption was observed when compared to control cells without insulin treatment. Furthermore, the molecular mechanisms leading to glucose homeostasis and/or IR are still uncertain. However, NLL γ-conglutin might be able to contribute in this process of glucose homeostasis, as we have demonstrated in the current study that glucose uptake by IR-C cells is clearly induced by treatment with γ-conglutin protein, reaching higher glucose uptake levels after IR-C cells challenged with 25 μg of γ-conglutin protein, which glucose uptake increased more than 60% in comparison to IR-C cells (*p* < 0.05), which were assayed without γ-conglutin protein challenge (Figure 4C). 

**Figure 5:**
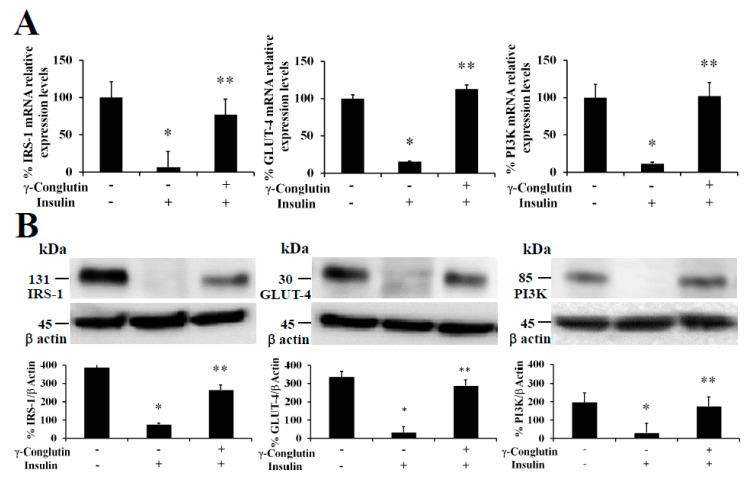
NLL γ-conglutin increases mRNA expression and protein levels of the insulin signaling pathway mediators IRS-1, PI3K and GLUT-4. PANC-1 cells or IR-C cell culture were incubated for 24 h alone, or the former culture with γ-conglutin. (**A**) The bar graph shows mRNA levels determined by real-time RT-qPCR of IRS-1, PI3K and GLUT-4. (**B**) The bar graph shows protein levels determined by immunoblotting of IRS-1, PI3K and GLUT-4. Average value from triplicate experiments of each biomarker were relativized to the average value of their housekeeping actin protein in control samples. Then, average values from challenge experiments (calculated in the same way than controls) are relativized to these from their respective control values previously calculated. Data represent mean ± SD from three independent experiments. Control: Untreated control PANC-1 culture cells; IR-C: insulin resistant culture cells; IR-C + γ: IR-C + γ-conglutin challenge. *p* < 0.05 represents statistically significant differences associated with each figure. p* < 0.05 IR-C *versus* control PANC-1 cells; p** < 0.05 IR-C + γ-conglutin *versus* IR-C. Challenges were made with 25 μg of γ-conglutin.

The treatment of pancreatic IR-C cells with γ-conglutin was also accomplished to determine whether this protein had effects on insulin resistance improvement throughout recovering the control-like associated mRNA expression levels of IRS-1, GLUT-4, and PI3K, key upstream and glucose transport mediators in the insulin signaling pathway [20], which would also be the reflect of a potential improvement in the glucose uptake and the inflammatory state on IR-C cells. The analysis of IRS-1, GLUT-4 and PI3K showed their up-regulation in their mRNA expression after γ-conglutin treatment in IR-C cells (Figure 5) [IRS-1: +70; GLUT-4: +97%; and PI3K: +90-fold, respectively], which differences were statistically significant compared to IR-C untreated cells (*p* < 0.05) (Figure 5A), as well as the mRNA expression level reduction of IRS-1, GLUT-4 and PI3K in IR-C cells [IRS-1: −93; GLUT-4: −84%; and PI3K: −89-fold, respectively] compared to control cells PANC-1 (Figure 5A).

We have also demonstrated that NLL γ-conglutin can reverse this state by up-regulating the IRS-1, GLUT-4 and PI3K functional protein levels in IR-C cells [IRS-1: +266; GLUT-4: +185; and PI3K: +144-fold, respectively] (Figure 5B), after decreased proteins levels showed when PANC-1 control cells acquired the IR-C statement compared to the control group [IRS-1: −302; GLUT-4: −310; and PI3K: −166-fold, respectively] (Figure 5B). These results confirm that γ-conglutin protein would be capable to reduce significantly the blood glucose level by promoting glucose uptake by insulin sensitive tissues while ameliorating hyperglycemia via increasing GLUT-4 glucose transporter protein level and plasma membrane recruitment [34], and insulin signaling pathway upstream mediators IRS-1 and PI3K [20].

Furthermore, at the same time we also evaluated the capability of γ-conglutin protein to regulate the mRNA and protein levels of pro-inflammatory molecules as potential mechanism helping to reverse the IR-C cell statement. TNFα, IL-1β and iNOS were analyzed in IR-C culture (Figure 3). These pro-inflammatory mediators were significantly lowered in γ-conglutin protein treated IR-C cells, at the mRNA expression levels [TNFα: −158; IL-1β: −144; and iNOS: −164-fold, respectively, *versus* IR-C untreated cells] (Figure 3A), and at the protein levels [TNFα: −189; IL-1β: −146; and iNOS: −97-fold, respectively, *versus* IR-C untreated cells] (Figure 3B, Supplementary Table S6). No statistically significant differences (*p* > 0.05) were found for TNFα, IL-1β and iNOS levels in IR-C cells treated with γ-conglutin in comparison to the PANC-1 control group (Figure 3). These results highlight the potential implications of γ-conglutin to improve insulin resistance through inflammation amelioration at molecular level in PANC-1 pancreatic cells by decreasing cytokines and iNOS levels [20].

In this study, we have demonstrated for the first time that NLL γ-conglutin protein is able to help improving the insulin resistance state in PANC-1 cell line targeting two major molecular signaling cross-roads, restoring functional levels of insulin activation pathway mediators while decreasing several pro-inflammatory mediators’ levels that worthwhile reinforces the first effect on PANC-1 cells. These outcomes are vital knowledge to be considered for successful anti-inflammatory insulin sensitizing new alternative therapies from natural plant sources.

### 3.6. Oxidative Stress Modulation by γ-Conglutin Protein as Anti-Inflammatory and Insulin Resistance Improvement Mechanism 

Oxidative stress, understood as the cellular statement of excess reactive oxygen species (ROS) production, is a main factor in the T2DM development [35], through promoting IR development. Afterward, high amounts of blood glucose sustained long time causes damage on the enzymes superoxide dismutase (Cu/Zn-SOD), catalase (CAT), and glutathione molecule as the most important elements of the cell antioxidant defense system [36]. Thus, an excessive ROS production contributes to oxidative stress, a pro-inflammatory state, and mitochondrial dysfunction that in turn exacerbates IR [37]. It would be necessary a comprehensive knowledge about the relationship between oxidative stress and T2DM risk factors (inflammation and IR) in order to improve diabetes prevention and its associated complications. In this regard, signaling molecules as nitric oxide (NO) play a critical role of the inflammation pathogenesis acting as a pro-inflammatory molecule, together with cytokines and chemokines (e.g., TNFα, IL-6, IL-12), under oxidative stress situations because of the excessive NO and ROS production, i.e., IR [38], promoting islet β-cell apoptosis [39] and the progression of diseases concomitant with inflammation [40].

**Figure 6:**
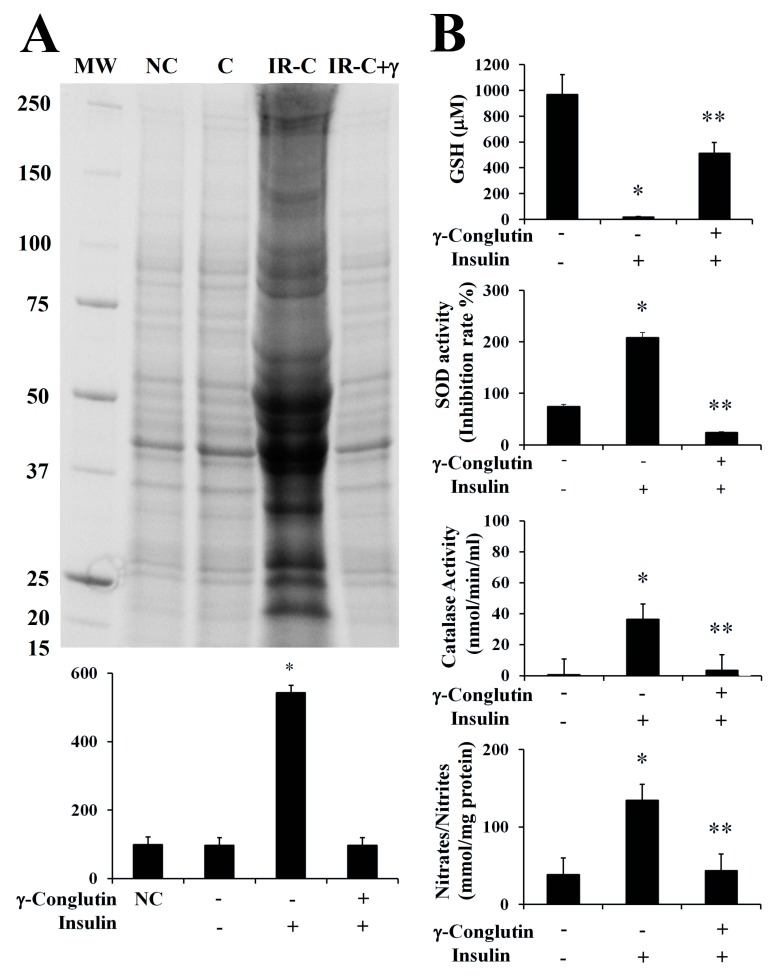
Effect of γ-conglutin on proteins oxidative modifications, antioxidant enzymatic activities and production of glutathione (GSH) and NO. (**A**) Changes in protein carbonyl formation were measured in IR-C cells after 24 h of incubation with γ-conglutin. Protein carbonyls were measured using an OxyBlot kit. Representative blots show basal carbonylation levels in C control PANC-1 cells, IR-C cells, and IR-C culture cells challenged with γ-conglutin. Graph y-axis represents arbitrary densitometry units. p* < 0.05 IR-C cells *versus* C cells. (**B**) IR-C pancreatic cells were incubated for 24 h with γ-conglutin protein. GSH and NO production, as well as SOD and catalase activities were measured. Data represent mean ± SD from three independent experiments. *p* < 0.05 represents statistically significant differences associated with each figure. p* < 0.05 IR-C *versus* control PANC-1 cells; p** < 0.05 IR-C + γ-conglutin *versus* IR-C. Challenges were made with 25 μg of γ-conglutin.

In the present study, we evaluated the oxidative homeostasis in inflammatory LPS-induced PANC-1 cells, as well as in IR-C cell model, after treatment with γ-conglutin protein. In both cases, we assessed the ROS production by measuring the levels of protein carbonylation, the covalent modifications of proteins induced by ROS, i.e., H_2_O_2_ or other derived molecules from the oxidative stress process by using an OxyBlot protein oxidation detection and immunoassay [41], and comparing them with control cells, LPS treated cells and IR-C cells, respectively, without any challenge with γ-conglutin. Very low levels of protein oxidation, generated through normal metabolic activity, were observed in untreated (control) cells with LPS (Supplementary Figure S3A), as well as in control PANC-1 cells before IR-C statement induction (Figure 6A). However, ROS production was significantly increased (*p* < 0.05) after LPS cells treatment (+677-fold, Supplementary Figure S3A), and in IR-C cells (+445-fold, Figure 6A), as significant (*p* < 0.05) increased levels of proteins carbonylation was detected. Treatments of these type of cells with γ-conglutin protein restored oxidative balance in both situations (LPS-induced cells: −423-fold, and IR-C cells: −445-fold, respectively; Supplementary Figure S3A, Figure 6A), in comparison to their respective inflammatory induced stages. These results suggest that γ-conglutin protein efficiently avoid at certain levels the ROS production (oxidative stress) in PANC-1 cells after inflammatory statement incensement, and that γ-conglutin exhibited strong anti-oxidant effect since this protein ameliorated the oxidative stress induced by LPS and in IR-C cell model. Interestingly, the present and future related studies would benefit from the comparative further analyses using other types of cell cultures, like primary islets and/or pancreatic β-cells and/or adipocyte cells to determine actions related to insulin secretion and islet inflammation.

Therefore, removal of free radicals is strongly dependent of enzymatic activities as superoxide dismutase (Cu/Zn-SOD), catalase (CAT) and glutathione (GSH) levels, representing crucial indicators of the cellular anti-oxidant capacity, and the oxidative stress cell state [35]. In the current study, we assessed the modulation of these antioxidant factors by γ-conglutin in the inflammatory LPS-induced PANC-1 cells, as well as in IR-C cell model, by measuring SOD and catalase activities, GSH levels and NO production, before and after the treatment with γ-conglutin (Supplementary Figure S3B, Figure 6B). We found a statistically significant (*p* < 0.05) decreased levels of GSH (LPS-induced inflammation cells: −660-fold; IR-C cells: −949-fold, respectively) (Supplementary Figure S3B, Figure 6B). Furthermore, the levels of SOD and catalase activity were strongly reduced after the same treatments with γ-conglutin protein in LPS-induced inflammatory statement (SOD: −677-fold; catalase: −142-fold, respectively) (Supplementary Figure S3B) and IR-C cells (SOD: −183-fold; catalase: −33-fold, respectively) (Figure 6B). These data showed that high GSH and low SOD levels and catalase activities might be regulated by γ-conglutin protein through direct or indirect marked effects in avoiding lipids and protein oxidative modifications, which is also supported by the concomitant large reduction of oxidative carbonylation (Supplementary Figure S3B, Figure 6B), and an overall oxidative stress balance improvement, translated also to an inflammation molecular cellular statement amelioration by γ-conglutin protein as an anti-oxidant protein. 

Furthermore, we analyzed the NO production again in both induced inflammation cell models treated with γ-conglutin protein for 24 h. Statistically significant decreased levels of NO were found (*p* < 0.05) in the LPS-induced cells (−351-fold, Supplementary Figure S3B) and IR-C cells (−91-fold, Figure 6B), in comparison to inflammation induced cells without γ-conglutin protein treatment, showing again how γ-conglutin is able to ameliorate the inflammatory state of cells promoting lowering NO [42] and iNOS expression levels, showing potential uses in the improvement of T2DM and other inflammatory-based diseases.

These novel results clearly indicated that oxidative stress is a major point targeted by NLL γ-conglutin protein effects causing an improved stress balancing through reduced ROS-related pro-inflammatory mediators and increased anti-oxidative molecules. Indeed, such data can be helpful for the development of future antioxidant and new anti-inflammatory therapeutics avoiding the oxidative stress activation of inflammatory mediators involved in several chronic diseases, with the advantage of being a natural product from lupin seeds that can be implemented as a functional food. 

## 4. Conclusions

In this study, treatment with NLL γ-conglutin protein to inflammation LPS-induced and IR-C in the PANC-1 pancreatic cell-line promoted: (i) Lowering expression of mRNA and proteins levels of key pro-inflammatory mediators as TNFα, IL-1β, and iNOS; ii) the up-regulation mRNA expression and increasing protein levels of IRS-1, and p85-PI3K, and GLUT-4 transporter, which are crucial biomarkers of the insulin signaling pathway activation. This up-regulation makes possible the recovery of the physiological condition of the cells as control cell-like situation from an induced inflammatory statement; (iii) glucose uptake in IR-C cells; (iv) a significant decrease (*p* < 0.05) in proteins levels of pro-inflammatory mediators INFγ, IL-6, IL-12, IL-17 and IL-27; (v) significant dropping oxidative stress in inflammation LPS-induced and IR-C pancreatic cells, as indicated by a reduced levels of protein carbonylation, improved glutathione (GSH) levels and lower SOD and catalase antioxidant enzymatic activities; (vi) reduction of NO production and down-regulation of iNOS in both, LPS-induced inflammation and IR-C pancreatic cells. This study is the first describing the anti-inflammatory effects at molecular level of the legume protein family 7S basic globulins or γ-conglutin, constituting strong evidences that NLL γ-conglutins play a crucial role in the development of novel functional foods and therapeutic options for the prevention and treatment of inflammatory-related diseases. 

## Supplementary Materials

The following are available online at https://www.mdpi.com/2076-3921/9/1/12/s1, Figure S1: Isolation and purification of NLL seed γ-conglutin protein; FigureS2: Effect of NLL γ-conglutin on the protein levels of pro-inflammatory cytokines; Figure S3: Effect of γ-conglutin on proteins oxidative modifications, antioxidant enzymatic activities and production of GSH and NO; Table S1: γ-conglutin peptides mass fingerprinting characterization; Table S2: Cell viability (%) and dose effects of NLL γ-conglutin protein; Table S3: Cell viability (%) on insulin resistance IR-C cell model; Table S4: Cell viability (%) and dose effects of purified NLL γ-conglutin protein on insulin resistance cell (IR-C) model; Table S5: Fold-change in protein levels of pro-inflammatory cytokines and iNOS; Table S6: Fold-change in protein levels of pro-inflammatory cytokines and iNOS.
