# Supplementary material for: Narrow-Leafed Lupin (Lupinus angustifolius L.) Seeds Gamma-Conglutin is an Anti-Inflammatory Protein Promoting Insulin Resistance Improvement and Oxidative Stress Amelioration in PANC-1 Pancreatic Cell-Line
Source: Antioxidants (Basel). 2019 Dec 23;9(1):12. doi: 10.3390/antiox9010012 (PMC7023629; doi:10.3390/antiox9010012)
Supplement: Supplementary file 1 [file antioxidants-09-00012-s001.pdf]

SUPPLEMENTARY MATTERIAL

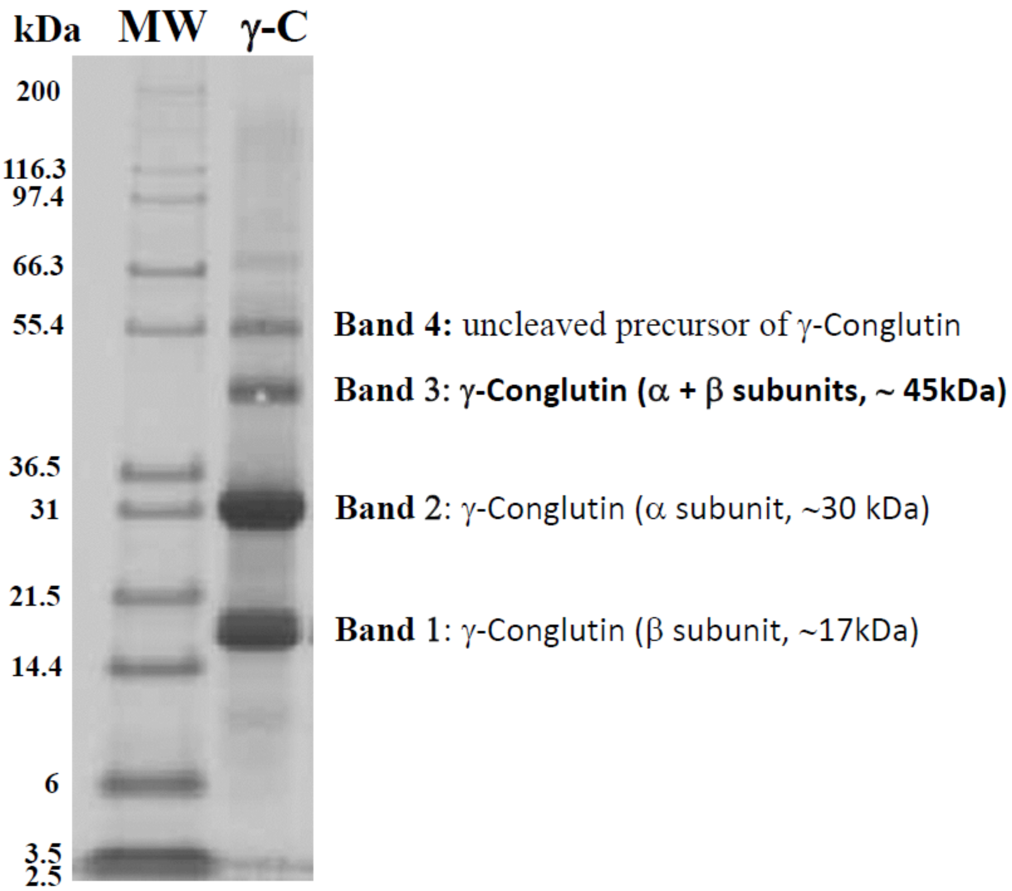

Figure S1. Isolation and purification of NLL seed γ-conglutin protein. SDS-PAGE of isolated and purified γ-conglutin protein, with high level of purity (>95%). Subunits α and β of γ-conglutin, unreduced γ-conglutin, and uncleaved γ-conglutin precursor are present. MW, molecular weight standard (kDa).

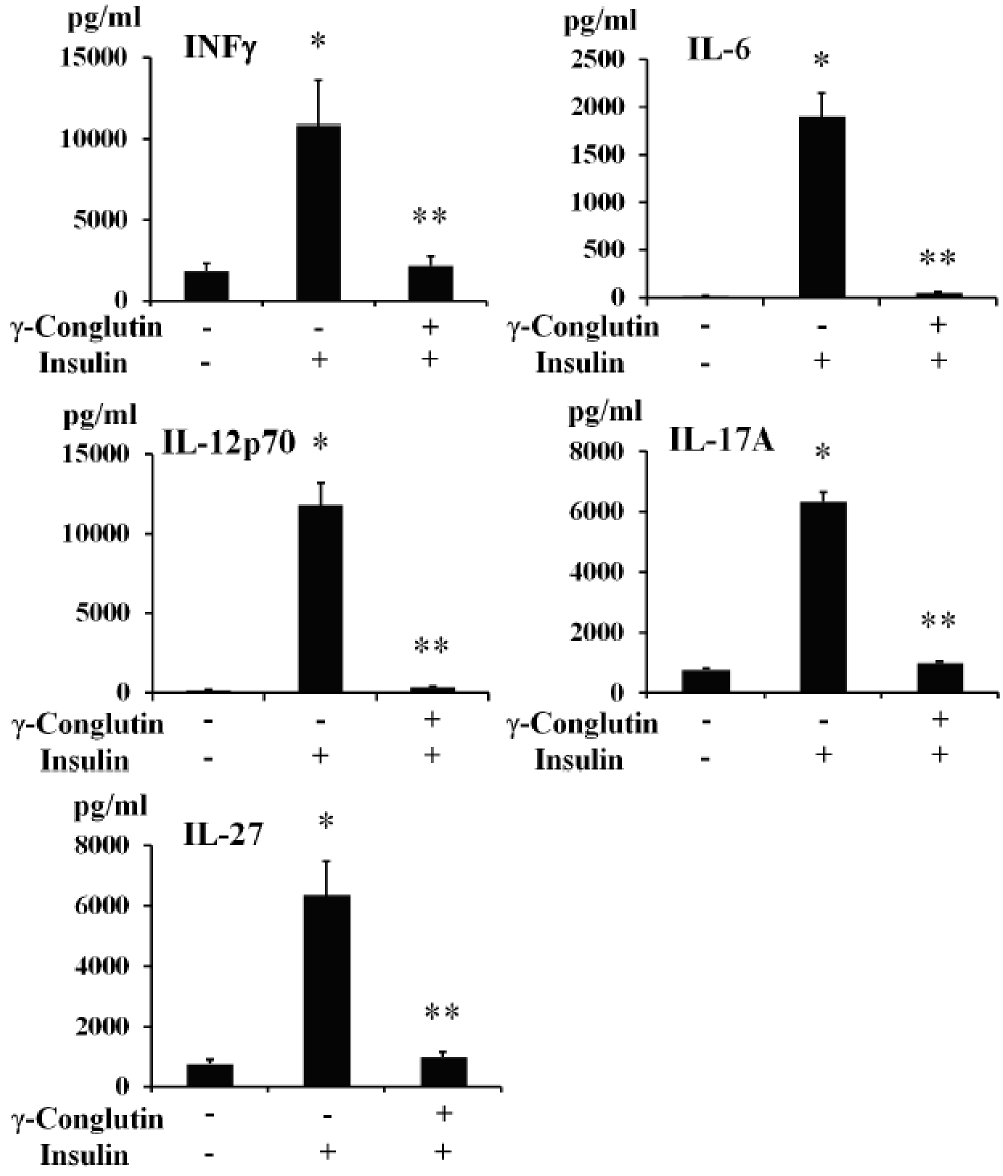

Figure S2. Effect of NLL γ-conglutin on the protein levels of pro-inflammatory cytokines. Control PANC-1 cells, and IR-C pancreatic cells were cultured for 24 h alone, or the former with γ-conglutin protein. The bar graph shows protein levels determined by ELISA of INFγ, IL-6, IL-12, IL-17, and IL-27. Data represent mean ± SD from three independent experiments. C: Untreated control culture cells; IR-C: insulin resistant culture cells; IR-C+γ: IRC+γ-conglutin challenge. $p < 0.05$ represent statistically significant differences associated with each figure. $p^* < 0.05$ IR-C *versus* C; $p^{**} < 0.05$ IR-C+γ-conglutin *versus* IR-C.

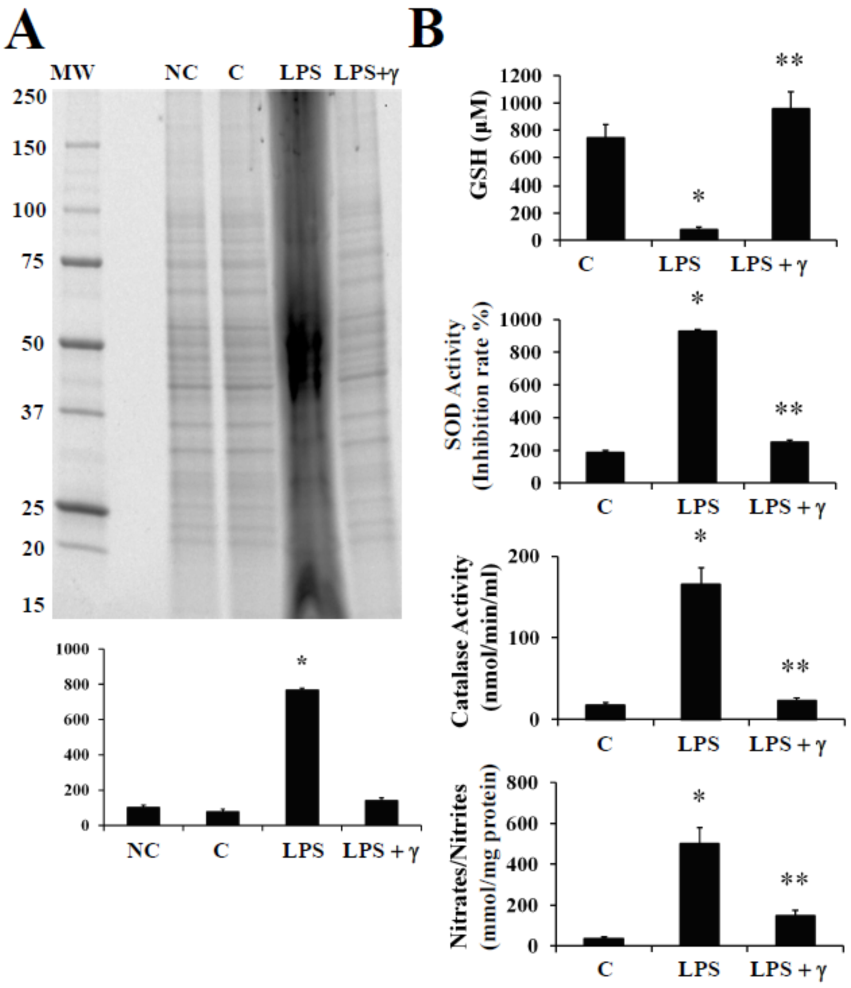

Figure S3. Effect of γ-conglutin on proteins oxidative modifications, antioxidant enzymatic activities and production of GSH and NO. (**A**) Changes in protein carbonyl formation were measured in LPS-treated pancreatic cells after 24 h of incubation with LPS and/or γ-conglutin. Protein carbonyls were measured using an OxyBlot kit. Representative blots show basal carbonylation levels in C control PANC-1 cells, LPS-treated pancreatic cells, and LPS-treated pancreatic cells challenged with γ-conglutin. Graph y-axis represents arbitrary densitometry units. $p^* < 0.05$ LPS-treated pancreatic cells *versus* C cells. (**B**) LPS-treated pancreatic cells were incubated for 24 h with γ-conglutin protein. GSH and NO production, as well as SOD and catalase activities were measured. Data represent mean ± SD from three independent experiments. $p < 0.05$ represent statistically significant differences associated with each figure. $p^* < 0.05$ LPS-treated pancreatic cells versus C (control PANC-1) cells; $p^{**} < 0.05$ LPS + -conglutin treated pancreatic cells versus LPS-treated pancreatic cells.

Supplementary Table S1. γ-conglutin peptides mass fingerprinting characterization.

Band[1]	Sequence of γ-conglutin protein[2]	identification	Sequence coverage (%)	Score[3]
1	MARNMAHILHILVISLSYSFLFVSSSSQDSQSLYHNSQPTSSKPNLLVLPVQEDASTGLH<br>WANIHKRTPLMQVPLLLDLNGKHLWVTCSQHYSSSTYQAPFCHSTQCSRANTHQCFTCTD<br>STTTRPGCHNNTCGLLSSNPVTQESGLGELAQDVLAIHSTHGSKLGPMVKVPQFLFSCAP<br>SFLAQKGLPNNVQGALGLGQAPISLQNQLFSHFGLKRQFSVCLSRYSTSNGAILFGDIND<br>PNNNNYIHNSLDVLHDLVYTPLTISKQGEYFIQVNAIRVNKHLVIPTKNPFISPSSTSYH<br>GSGEIGGALITTTHPYTVLSHSIFEVFTQVFANNMPKQAQVKAVGPFGLCYDSRKISGGA<br>PSVDLILDKNDAVWRISSENFMVQAQDGVSCLGFVDGGVHARAGIALGAHHLEENLVVFD<br>LERSRVGFNSNSLKSYGKTCSNLFDLNNP	Conglutin gamma *Lupinus angustifolius (Narrow-leaved blue lupine)*	26.3	90.2
2	MARNMAHILHILVISLSYSFLFVSSSSQDSQSLYHNSQPTSSKPNLLVLPVQEDASTGLH<br>WANIHKRTPLMQVPLLLDLNGKHLWVTCSQHYSSSTYQAPFCHSTQCSRANTHQCFTCTD<br>STTTRPGCHNNTCGLLSSNPVTQESGLGELAQDVLAIHSTHGSKLGPMVKVPQFLFSCAP<br>SFLAQKGLPNNVQGALGLGQAPISLQNQLFSHFGLKRQFSVCLSRYSTSNGAILFGDIND<br>PNNNNYIHNSLDVLHDLVYTPLTISKQGEYFIQVNAIRVNKHLVIPTKNPFISPSSTSYH<br>GSGEIGGALITTTHPYTVLSHSIFEVFTQVFANNMPKQAQVKAVGPFGLCYDSRKISGGA<br>PSVDLILDKNDAVWRISSENFMVQAQDGVSCLGFVDGGVHARAGIALGAHHLEENLVVFD<br>LERSRVGFNSNSLKSYGKTCSNLFDLNNP	Conglutin gamma *Lupinus angustifolius (Narrow-leaved blue lupine)*	18.4	60.2

Table S2. Cell viability (%) and dose effects of NLL γ-conglutin protein. Cytotoxicity of γ-conglutin protein on PANC-1-pancreatic cells. Cells were treated with 10, 25 and 50 μg of sample proteins for 24 h. Cell viability after treatment with LPS (1 μg) was 99.0 ± 1.4%. Data represent mean ± SD from three independent experiments. Controls were performed using PANC-1 cells without any treatment with LPS and/or γ-conglutin protein. $p < 0.05$ represent statistically significant differences associated with the table. $p^* < 0.05$ treated cells versus untreated control PANC-1 cells.

Samples	10 μg	25 μg	50 μg
γ-conglutin	97.2 ± 1.5	99.6 ± 2.4	82.0 ± 2.6*
LPS (1 μg) + γ-conglutin	97.6 ± 2.8	99.8 ± 2.4	81.0 ± 1.3*

Table 3. Cell viability (%) on insulin resistance IR-C cell model. PANC-1 cells were treated with increasing (10^{-9}, 10^{-8}, 10^{-7}, 10^{-6}, 10^{-5} nmol/L) insulin quantities. Data represent cell viability (%) mean ± SD from three independent experiments. $p < 0.05$ represent statistically significant differences associated with the table. $p^* < 0.05$ treated cells versus untreated control PANC-1 cells.

Insulin (nmol/L)	% viability
10^{-9}	99.5 ± 1.5
10^{-8}	99.4 ± 1.2
10^{-7}	99.1 ± 1.5
10^{-6}	95.4 ± 1.6*
10^{-5}	94.7 ± 1.8*

Table S4. Cell viability (%) and dose effects of purified NLL γ-conglutin protein on insulin resistance cell (IR-C) model. Cytotoxicity of γ-conglutin protein on IR-C pancreatic cells. Cells were treated with 10, 25 and 50 μg of sample proteins for 24 h. Data represent cell viability (%) mean ± SD from three

independent experiments. Control samples were assayed only with insulin (10^{-7} nm/L) showing 100% of cell viability. $p < 0.05$ represent statistically significant differences 6 associated with the table. $p^* < 0.05$ treated cells versus untreated control PANC-1 cells.

Sample	10 μg	25 μg	50 μg
γ-conglutin protein	98.6 ± 2.8	99.0 ± 1.8	80.0 ± 0.8*

Table S5. Fold-change in protein levels of pro-inflammatory cytokines and iNOS. Numbers represent fold-changes calculated versus LPS from data in Figures 1 and 2 for a) LPS-induced inflammation model; and versus IR-C from data in Figure 5 and Supplementary Figure S2 for b) IR model. Positive and negative values mean up-and down-regulated genes, respectively.

a)

Cytokines	γ-conglutin + LPS
TNFα	–253
INFγ	–12443
IL-1β	–146
IL-6	–1849
IL-12p70	–11792
IL-17A	–5339
IL-27	–6100
iNOS	–258

b)

Cytokines	γ-conglutin + IR
TNFα	–146
INFγ	–8644
IL-1β	–97
IL-6	–1839
IL-12p70	–11409
IL-17A	–5659
IL-27	–5339
iNOS	–189

Table S6. Fold-change in protein levels of pro-inflammatory cytokines and iNOS. Numbers represent fold-changes calculated versus control from data in Figures 1 and 2 for a) LPS-induced inflammation model; Figure 5 and Supplementary Figure S2 for b) IR model. Positive and negative values mean up- and down-regulated genes, respectively.

a)

Cytokines	LPS	γ-conglutin + LPS
TNFα	+145	-1
INFγ	+11335	-1100
IL-1β	+187	-66
IL-6	+2979	-119
IL-12p70	+12127	+341
IL-17A	+5632	-492
IL-27	+5676	+300
iNOS	+245	-13

b)

Cytokines	IR	γ-conglutin +IR
TNFα	+173	+18
INFγ	+8994	+350
IL-1β	+129	+27
IL-6	1881	+32
IL-12p70	+11592	+208
IL-17A	+5553	+214
IL-27	+5231	+218
iNOS	+287	+32
